# Residual cyst of the jaws: A clinico-pathologic study of this seemingly inconspicuous lesion

**DOI:** 10.1371/journal.pone.0244250

**Published:** 2020-12-17

**Authors:** Fadi Titinchi, Jean Morkel

**Affiliations:** Department of Maxillo-Facial and Oral Surgery, Faculty of Dentistry and WHO Collaborating Centre, University of the Western Cape, Tygerberg Oral Health Center, Cape Town, South Africa; Thamar University, Faculty of Dentistry, YEMEN

## Abstract

**Objectives:**

Residual cysts are relatively rare inflammatory cysts of the jaws. They are essentially radicular cysts without the presence of the offending dentition. These lesions have the ability to destroy bone within the jaws without any symptoms. Moreover, they can mimic more aggressive cysts and tumours on radiographs. The aim of this study was to describe the clinico-pathological features of residual cysts in order to discern them from other cystic lesions as well as analyse their management and recurrence patterns.

**Materials and methods:**

Sixty-four histopathologically confirmed residual cysts were analysed based on their clinical, radiological and histopathological features. Their management and follow-up were also noted.

**Results:**

The majority of lesions presented in elderly (46.8%), edentulous patients (60.9%) and were most commonly found in the posterior regions of the mandible (51.6%). Clinico-pathological features that aided in their diagnosis included long-standing history with slow growing swelling and presence of well-defined, unilocular cystic lesion associated with previously extracted dentition. Enucleation was a successful method in the management of residual cysts with very low recurrence rate (1.6%). Two patients (3.1%) developed squamous cell carcinoma from the cyst lining.

**Conclusion:**

Residual cysts should be high on the list of differential diagnosis when elderly, edentulous patients present with cystic lesions in the jaws compared to dentate patients (*P*<0.01). All lesions should by biopsied and sent for histopathological examination along with radiological correlation as they have the potential to transform into primary intra-osseous squamous cell carcinoma with devastating consequences to the patient.

## 1. Introduction

The jaws are the most frequently affected bones in the human body by cysts. This is due to the numerous epithelial rests that form a close relationship with developing dentition [1]. The clinico-pathological and radiographic presentation of these cysts in the jaws may resemble each other and hence many cysts may mimic tumours and intra-osseous lesions [1].

Cysts of the jaws are classified according to their origin into odontogenic and non-odontogenic cysts. Cysts of odontogenic origin are the most prevalent type and develop from the epithelium of developing dentition. The epithelium of these cysts may arise from the dental organ, rests of Malassez, reduced dental epithelium and fragments of dental lamina. These epithelial remnants may lead to the development of a residual cyst following the removal of the involved offending tooth [2].

Residual cyst is inflammatory in origin and is usually preceded by a radicular cyst in the jaws which has formed apical or adjacent to an extracted tooth [3]. Residual cysts usually present with the same features as those of conventional radicular cysts; however, due to removal of the cause (i.e. carious tooth/roots), the inflammatory infiltrate in these remnant cysts decreases and non-inflammatory fibrous collagen tissue is present in their walls [3]. These cysts also have a thin epithelial lining which makes their identification by histopathological methods more challenging [4]. Some studies reported that residual cysts show active growth patterns in areas which have been edentulous for several years [5]. The variable behaviour of these cysts highlights the importance of further detailed studies on these often overlooked lesions.

There is paucity in the literature regarding the clinico-pathologic presentation of residual cysts of the jaws [3, 6]. This is especially important as they may mimic more aggressive cysts and tumours of the jaws. They have also been reported to rarely transform into squamous cell carcinoma and even less is known about the success of different surgical methods in their management [3]. Hence the rationale for this study was to describe the presenting features of a large case-series of these cysts as well as their management and recurrence patterns.

## 2. Methods

This study was a retrospective case-series analysis of all histopathologically confirmed residual cysts presenting at a tertiary referral hospital in Cape Town, South Africa. Ethics approval was obtained from Biomedical Research Ethics Committee of the University of the Western Cape. Individual patient consent was waived by the ethics committee due to the retrospective nature of the study. The data recorded included the patient’s age, gender and ethnic background. The presenting signs and symptoms of the patient as well as the history of the lesion were collected. The detailed location of the lesion within the jaws was noted. Histopathological features were recorded including any cases that developed into squamous cell carcinoma. Management and follow-up was also noted.

The location of the lesion was classified into varying anatomical regions in the jaws. The anterior region of the mandible extended from the left canine to right canine and in edentulous patients from the left mental foramen to right mental foramen. The posterior region of the mandible extended from first premolar to the angle of the mandible, for both sides. The anterior region of the maxilla extended from the left canine to right canine while the posterior region extended from first premolar to the maxillary tuberosity on both sides.

The size of lesions on radiographs was measured in millimetres using the widest diameter from one end of the lesion to the other. The overall shape of the lesion was noted along with the radio-density and locularity. The margins were classified as either well-defined or ill-defined. The effect of the lesion on adjacent structures (adjacent dentition, mandibular canal and/or maxillary antrum) was recorded. Lesions were further categorised as either unilocular (only one compartment) or multilocular (multiple adjacent compartments within the cavity). The effect of the lesion on the cortex of the mandible was also noted to determine the expansive nature of the lesion. Signs of root resorption were documented to demonstrate the aggressive nature of the lesion.

Data was analysed using student’s t-test and chi-square test to draw correlations between various parameters such as lesion size, gender, locularity, radio-density etc. Epi Info V7 software was used to perform statistical analysis. Statistical significance was set at *P* < 0.05.

## 3. Results

### 3.1. Demographic and clinical findings

A total of 64 residual cysts were diagnosed in 62 patients over a period of 30 years. The frequency of residual cysts amongst all odontogenic cysts was relatively low (3.2%). The ages of patients ranged from as young as 11 years to 82 years (mean: 48 years). The majority of patients (46.8%) were above 50 years of age. Males (54.8%) were slightly more affected than females (45.2%).

Symptoms were present in 57.8% of patients which included swelling (64%), pain (25%) and pus discharge (6.3%). Only one patient presented with paraesthesia. Symptoms were present for a mean period of 6.8 months prior to presentation. Most symptomatic lesions occurred in the posterior mandibular regions (77.8%).

### 3.2. Radiological findings

The majority of lesions occurred in the mandible (73.4%) with the posterior regions being the most affected site (51.6%). Most lesions appeared as unilocular (95.3%), well-defined (93.7%) and had smooth margins (85.9%). Bony expansion was not a common feature while only one case caused root resorption. The lesion demonstrated the ability to cause displacement of adjacent anatomical structures including mandibular canal (45.7%) and maxillary antrum (66.6%). Summary of radiological finding is presented in Table 1.

**Table 1:** Summary of radiological findings of residual cysts of the jaws.

	Anterior Mandible	Posterior Mandible	Anterior Maxilla	Posterior Maxilla	Total
**Locularity**					
Unilocular	13 (20.3%)	32 (50%)	10 (15.6%)	6 (9.3%)	61 (95.3%)
Multilocular	1 (1.6%)	1 (1.6%)	0	1 (1.6%)	3 (4.7%)
**Demarcation**					
Well-defined	14 (21.8%)	31 (48.4%)	9 (14.1%)	6 (9.3%)	60 (93.7%)
Ill-defined	0	2 (3.2%)	1 (1.6%)	1 (1.6%)	4 (6.3%)
**Shape**					
Irregular	2 (3.2%)	15 (23.4%)	2 (3.2%)	4 (6.3%)	23 (35.9%)
Round	3 (4.7%)	9 (14.1%)	5 (7.8%)	1 (1.6%)	18 (28.1%)
Oval	9 (14.1%)	9 (14.1%)	3 (4.7%)	2 (3.2%)	23 (35.9%)
**Margins**					
Smooth	12 (18.7%)	29 (45.3%)	9 (14.1%)	5 (7.8%)	55 (85.9%)
irregular	2 (3.2%)	4 (6.3%)	1 (1.6%)	2 (3.2%)	9 (14.1%)
**Bony expansion**	5 (7.8%)	12 (18.7%)	2 (3.2%)	2 (3.2%)	21 (32.8%)
**Sclerotic margin**	13 (20.3%)	24 (37.5%)	6 (9.3%)	4 (6.3%)	47 (73.4%)
**Root resorption**	0	1 (1.6%)	0	0	1 (1.6%)

**Fig 1:**
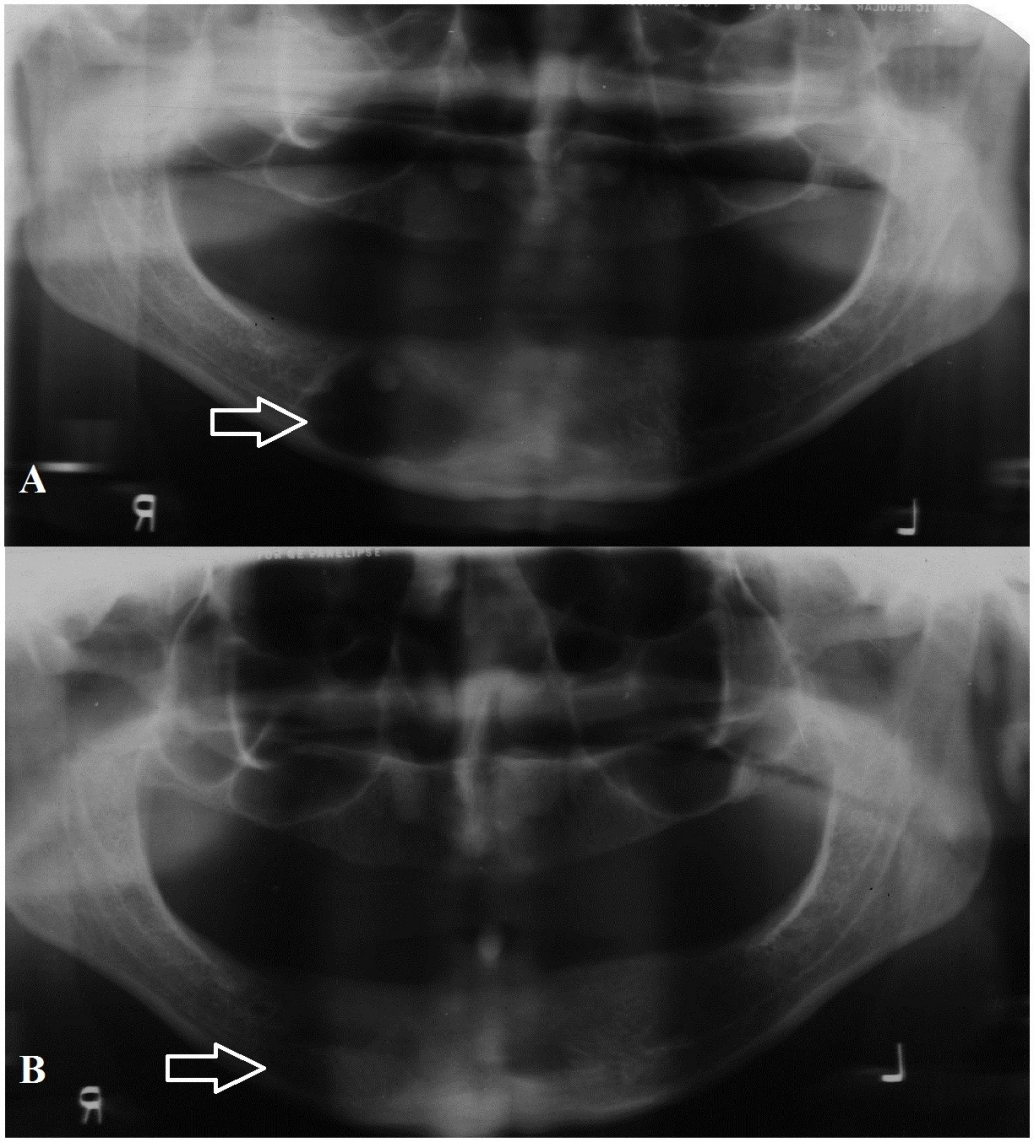
**A**: Panoramic radiograph showing incidentally found residual cyst in the mandible with calcification. **B**: Two year post-operative radiograph of same patient showing bone regeneration in the defect.

Interestingly, most patients in this sample were edentulous (60.9%). Seventeen lesions (26.5%) were incidentally found on routine panoramic radiographs (Fig 1). One-third of incidentally found lesions occurred in edentulous patients. These incidental lesions were significantly smaller in size than symptomatic lesions (*P* < 0.05).

Due to the high frequency of these cysts in elderly patients with radiological features that resemble other cystic lesions of the jaws, odontogenic keratocyst and ameloblastoma were high on the list of differential diagnosis in this sample. Only thirty (46.8%) of lesions were accurately diagnosed by the consulting surgeon based on clinical and radiological features alone.

### 3.3. Histopathological features

**Fig 2:**
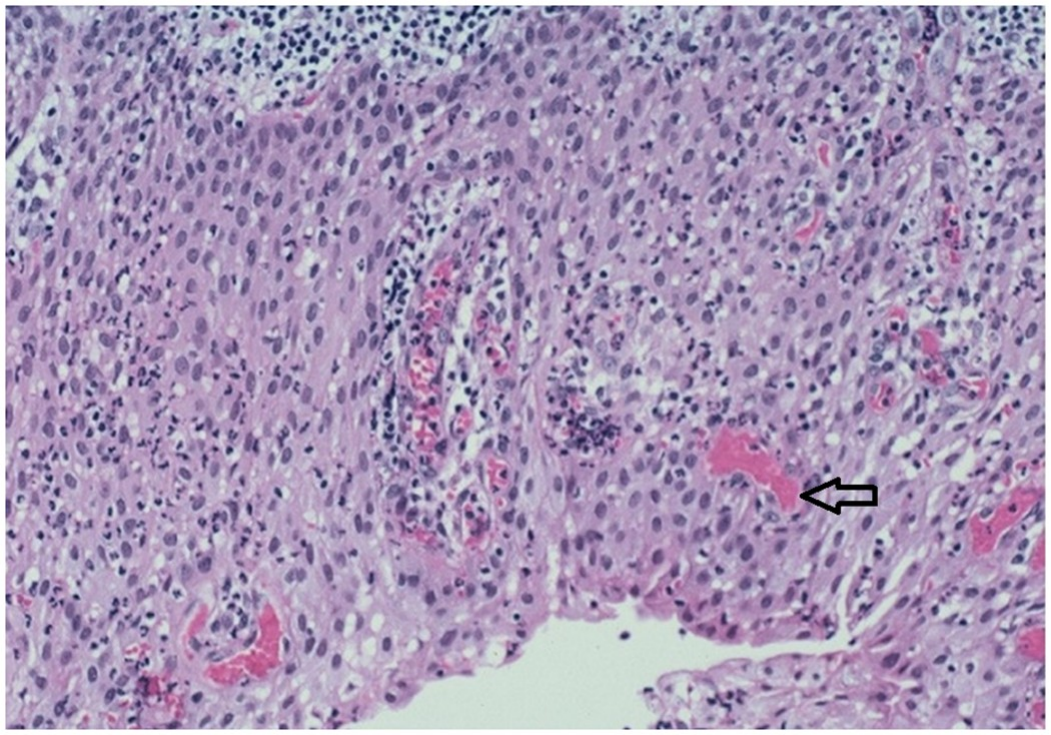
High power magnification of histological section (H & E staining) showing hyaline bodies in the epithelium with dense chronic inflammatory infiltrate.

**Fig 3:**
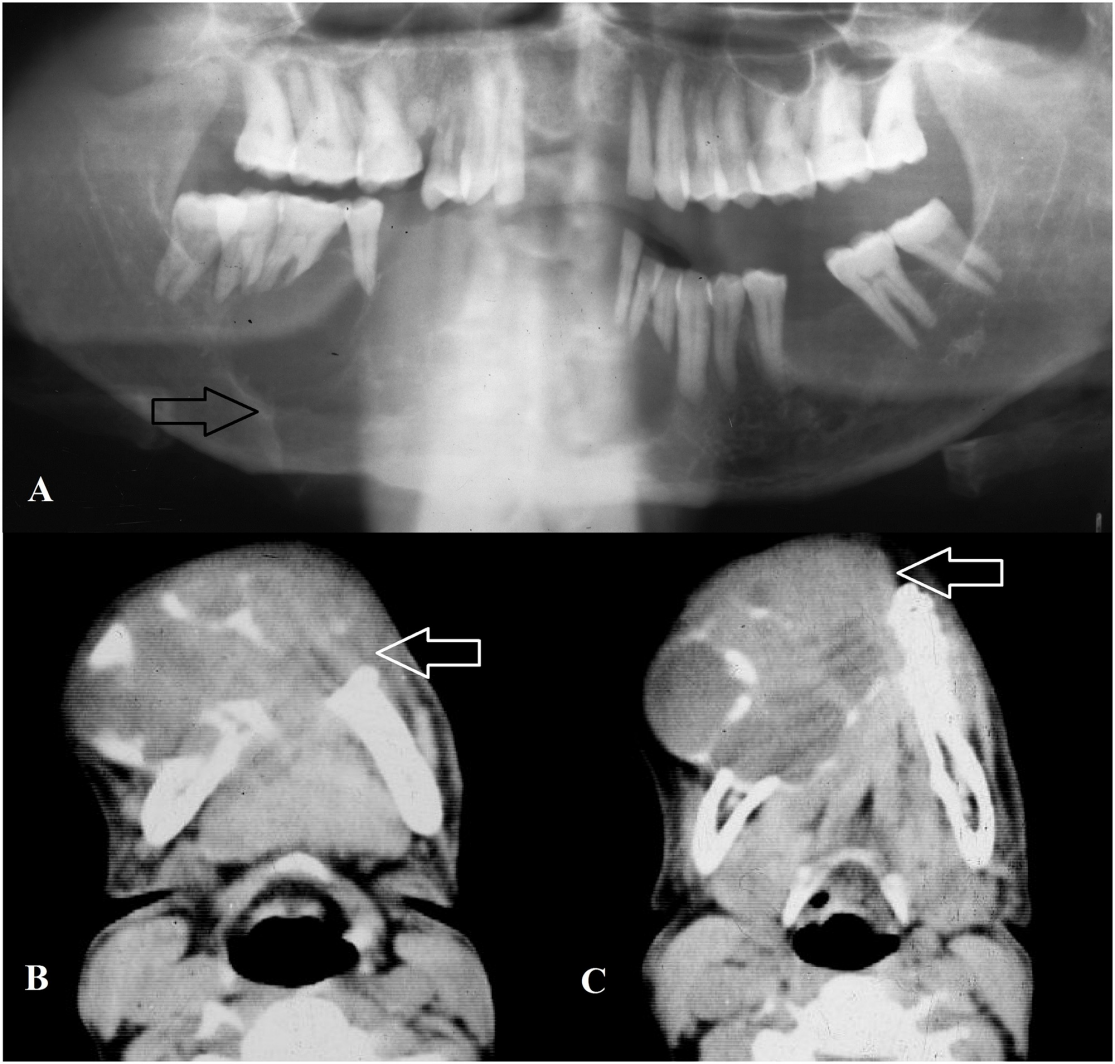
**A**: Panoramic radiograph showing destructive, ill-defined squamous cell carcinoma arising from the epithelial lining of residual cyst in the mandible. **B and C**: Axial computed tomography images of same patient showing invasive, expansive and multilocular lesion in the mandible.

All lesions showed a cyst lined by proliferative stratified squamous epithelium with some lesions showing foci of mucous metaplasia. Dense chronic inflammation was noted within the fibrous wall of the cyst (Fig 2). Cholesterol cleft was not a very prominent feature (39%) and only 5 lesions showed calcifications (7.8%). Two residual cysts showed signs of squamous cell carcinoma within their lining. Both lesions occurred in the mandible and caused extensive destruction with cervical lymph node involvement at the time of presentation (Fig 3).

### 3.4. Management and recurrence

Most lesions were surgically managed with enucleation of the cystic lining while only one case was initially marsupilized due to its extensive size followed by enucleation. Large lesions that underwent enucleation were packed with bismuth iodine paraffin paste (BIPP) impregnated gauze for an initial period of two weeks followed by incremental removal of BIPP weekly over two to three visits depending on the size of the defect. This aided in eliminating the dead space and avoided the need for bone grafting. Both patients with malignancy in the cyst lining underwent resection with one patient demising 8 months later.

**Fig 4:**
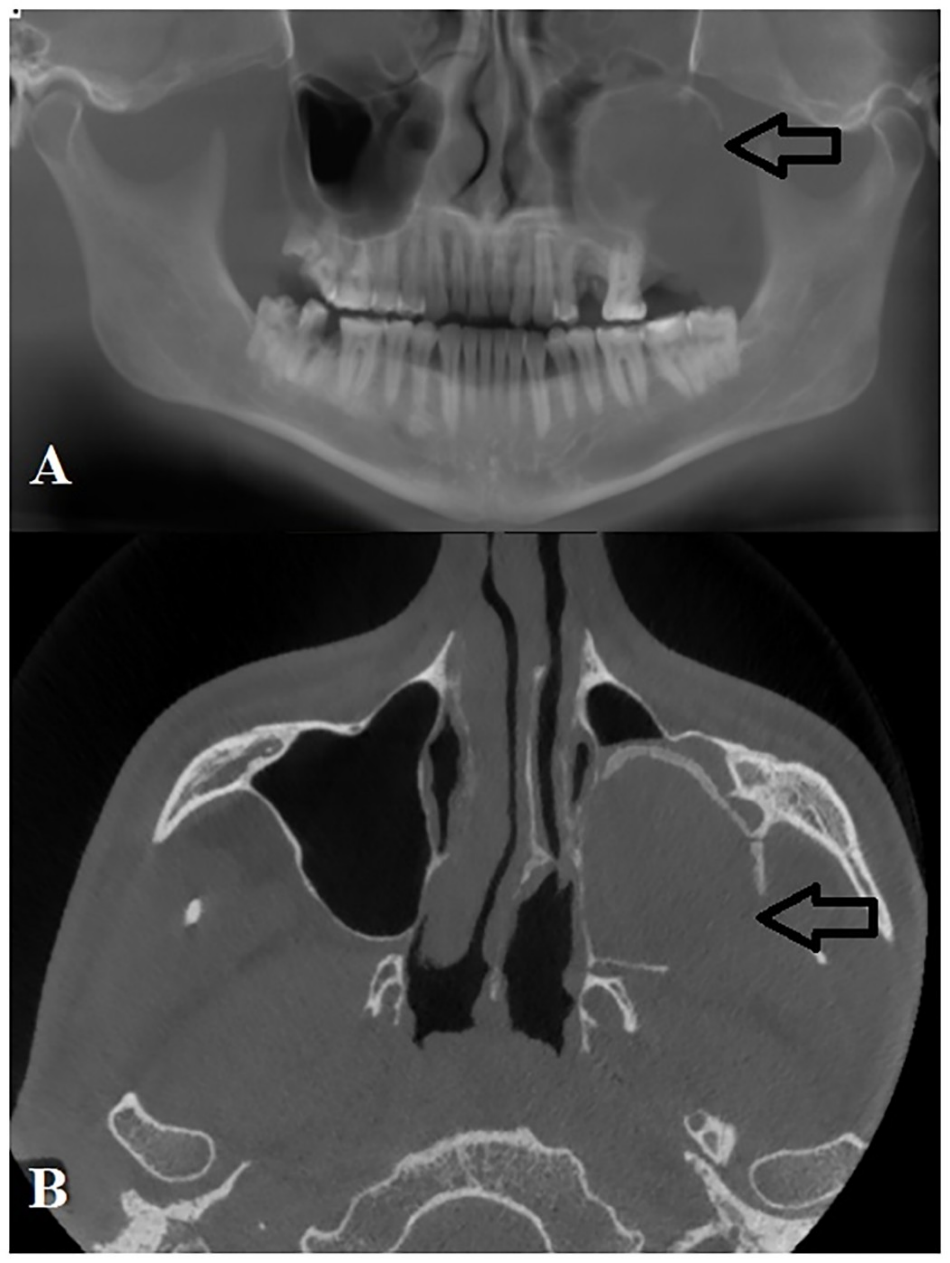
**A**: Panoramic reconstruction of cone beam computed tomography (CBCT) images of large residual cyst in the left maxilla that recurred following enucleation due to extension into maxillary antrum. **B**: Axial view of same patient showing residual cyst occupying the entire left maxillary antrum.

**Fig 5:**
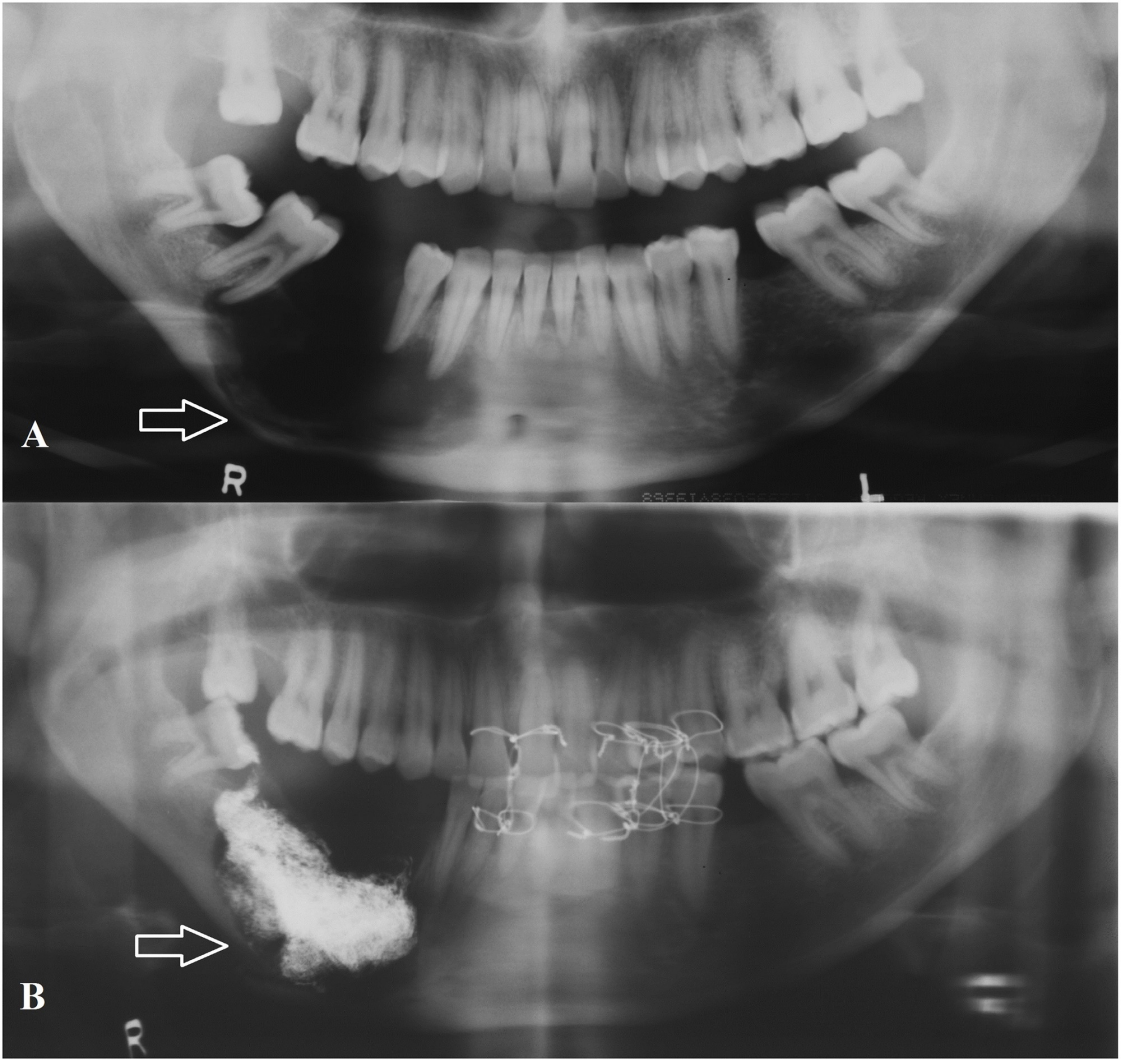
**A**: Pre-operative panoramic radiograph of residual cyst in the mandible with notable thinning of the mandibular cortex. **B**: Post-operative panoramic radiograph of same patient showing pathological fracture of mandible with BIPP in situ to prevent post-operative sepsis.

Although residual cysts are not known to recur, one patient who underwent enucleation of cystic lesion in maxilla returned with recurrent lesion 5 months post-operatively (Fig 4). This was most likely due to inadequate enucleation of the lesion which extended into the maxillary antrum. Other adverse complications include one patient who suffered a pathological fracture of the mandible post enucleation of the lesion (Fig 5).

## 4. Discussion

Residual cysts are often overlooked and assumed to be unambiguous in its presentation. For this reason, there are only a handful of detailed case series in the literature. This is one of the largest detailed studies in the literature on residual cysts. It highlights its variable clinico-pathological presentation and its ability to continually grow undetected mimicking more aggressive cysts and tumours.

Inflammatory cysts are the most frequent type of cysts in the jaws. This group of cysts comprise of radicular and residual cysts of which residual cysts are the second most common. The frequency of residual cysts have been reported to range from 2.2%–18% of odontogenic cysts in the jaws [2, 4] (Table 2). In this sample, the frequency was relatively low due to high rates of early extractions of carious teeth which prevented progress into inflammatory cysts.

**Table 2:** Frequency and demographics of residual cysts in the jaws in the literature.

Author	Region	Frequency	Ranking amongst odontogenic cyst	Mean age range (decade)	M:F ratio	Maxilla:Mandible ratio
Present study	South Africa	3.20%	4th	4th-5th	1.2:1	1:2.8
Nuṅez-Urrutia et al. [20]	Spain	4.30%	4th	3rd-4th	NA	1:1
Kambalimath et al. [9]	India	6.00%	5th	6th	3.5:1	NA
Ledesma- Montes et al. [21]	Mexico	4.90%	4th	3rd	0.88:1	NA
Ochsenius et al. [22]	Chile	11%	4th	5th-6th	1.15:1	2:1
Acikgoz et al. [23]	Turkey	13.70%	3rd	NA	NA	1:0.85
Khosravi et al. [8]	Iran	12.98%	3rd	3rd	1.77:1	1:1.06
Avelar et al. [24]	Brazil	5.91%	3rd	2nd	NA	1:1.16
Mosqueda-Taylor et al. [25]	Mexico	2.20%	4th	NA	NA	NA
Hu and You [26]	Korea	NA	NA	3rd-4th	1.23:1	2.3:1

NA: not available.

The pathogenesis of radicular and residual cysts is similar [3]. The process is initiated by the spread of bacteria from a non-vital tooth in the periapical region of the jaw. If untreated, this infection leads to the formation of a periapical granuloma which contains activated T cells that produce cytokines. These cytokines act on the epithelial remnants leading to proliferation of these remnants and differentiation into cyst formation [3]. The proliferating epithelium becomes oedematous through accumulation of fluid and coalesces to form microcysts lined by epithelial cells with inflammatory infiltrate. The cyst wall has a semi-permeable membrane and hence through osmosis, the cyst increases in size. Furthermore, lytic by-products from epithelial and inflammatory cells raise the osmotic pressure within the cyst leading to further expansion and formation of large intra-osseous cystic lesion within the jaws [3].

Residual cysts primarily affect middle-aged patients in the third decade of life but can manifest from as early as second to the eighth decades of life [7]. In this study, patients were older than previously reported most likely due to limited access to advanced dental imaging in community clinics which delays the diagnosis of these lesions. Most authors report that the lesion has slightly higher predilection for males as was shown in this sample [3, 8]. Kambalimath et al. [9] reported in their sample that males are 3.5 times more commonly affected than females.

Residual cysts in the jaws are usually asymptomatic and can be detected incidentally on routine radiography [3]. However, if the cyst becomes secondarily infected, patients may report pain and swelling and become aware of the lesion. As the cyst gradually increases in size, they can cause tooth displacement and mobility [2]. More than half of patients in this sample were symptomatic with swelling being the most common symptom. These symptoms are non-specific as other odontogenic lesions can have similar presentation but they should lead the clinician to investigate further.

High and Hirschmann reported in their sample that symptomatic cysts were more commonly found in the anterior maxilla, with a frequency of 37% [10]. This is due to the high prevalence of palatal invaginations and trauma which can lead to pulpal necrosis in the dentition in this region. Our study was in disagreement with this finding as most symptomatic lesions occurred in the posterior mandible. This further complicates distinguishing this cyst from other odontogenic cysts and tumours as they also have a high tendency to occur in this region.

Residual cysts in edentulous areas can cause expansion of the alveolar ridge preventing prosthetic restoration of the affect area caused by the resultant bony defect [2]. The majority of patients in this sample were indeed edentulous which reported significant reduction in their quality of life due the bony expansion caused by these cysts. Interestingly, Bodner et al. [11] reported that the majority of cystic lesions in edentulous jaws were residual cysts. Hence, based on the evidence presented in this study and other studies, residual cysts should be high on the list of differential diagnosis of cystic lesions in edentulous jaws.

Residual cysts present as unilocular, well-defined, radiolucent lesions on radiographs. They are usually round or oval in shape with a thin sclerotic border [3]. This fairly predictable radiological presentation was also noted in this study. These cysts can also cause significant bone resorption and displacement of vital areas such as inferior alveolar canal, maxillary antrum and nasal cavity as demonstrated in this study [2].

In most studies from Europe, the maxilla is reported to be more commonly affected than the mandible [2, 4]. In this sample, the mandible was overwhelmingly more involved than the maxilla. This finding is explained by the mechanism of tooth loss where by in Europe most causes of tooth loss are accidental injury to the maxillary incisors whereas in developing countries, dental caries is the main instigating factor for development of these cysts, which usually occurs in the posterior regions of the jaws.

Histological examination demonstrates that the cyst is usually lined by squamous epithelium. In newly formed cysts, the epithelial lining can exhibit signs of proliferation and arcading with marked inflammatory infiltrate but as the cyst gradually enlarges, the lining becomes dormant and consistent with varying amount of differentiation to mimic stratified squamous epithelium [2]. The histopathological features of these cysts in this study are consistent with these finding.

Calcifications in residual cysts are rarely reported as shown in this sample. These calcifications usually present in long-established lesions and those with chronic inflammation. The calcified masses may be barely noticeable, fine grains of radio-opacity, or large irregular particles. Dystrophic calcifications occur due to the deposition of calcium salts in areas of chronic inflammation or in necrotic tissues [10, 12]. These calcifications further complicate the diagnosis of these cysts as a number of other odontogenic lesions may have similar presentation such as calcifying odontogenic cyst, dentinogenic ghost cell tumour and calcifying epithelial odontogenic tumour.

Histopathological examination remains the main method of diagnosis of these lesions along with radiological correlation. However, heavily inflamed odontogenic cysts pose major diagnostic challenges as the inflammatory changes disguises the true features of the cyst on histological specimen [13]. In these cases, clinical and radiological correlation along with histochemical and molecular markers becomes vital to arrive at an accurate diagnosis [13].

Residual cysts are usually surgically managed by enucleation, marsupialization or decompression to decrease the intraluminal pressure within the cyst [14]. Smaller lesions can be enucleated entirely at the time of biopsy and is the ideal treatment choice. Enucleation was shown to be an acceptable method in this sample with low morbidity and recurrence rate. However, there are some contra-indications to enucleation which include large lesions, difficult accessibility, proximity to adjacent vital structures, and patient’s age. Hence, high-risk patients, younger individuals and the elderly should be managed with minimally invasive procedures to reduce morbidity [15].

Larger residual cysts particularly those extending into adjacent anatomical structures should be managed with marsupialization over a period of time to reduce the size of the cyst followed by enucleation of the remaining cyst lining [2]. This method was used for one extensive lesion in this sample that extended into the ramus of the mandible. A major disadvantage of this technique is that it requires multiple procedures under general anaesthesia.

Decompression is another treatment option that reduces intra-cystic pressure. This technique involves making a smaller window in the cyst wall, which is kept patent by suturing a device to it. Although both marsupialization and decompression are based on the principle of releasing intramural pressure to shrink the cyst size; at the same time they allow gradual bone deposition around the lesion. Decompression is regarded as the more appropriate treatment option in patients that warrant a more conservative approach [16].

Residual cysts have low recurrence rate following enucleation hence the prognosis following surgical management is good. If during surgical removal the cystic lining and wall is severely fragmented, leaving epithelial rests in the cavity, a residual cyst may recur in the area after a period of time as was the case in one of the patients in this study [17]. Therefore, periodic follow-up is essential to detect any recurrences and malignant transformation [12]. No previous study has reported on the recurrence rate of residual cysts in the literature and hence no comparison could be made with the recurrence rate of 1.6% in this sample.

Following surgical intervention, the defect gradually fills with bone however in certain cases, where the mandibular cortex is involved in an edentulous patient, then pathological fracture can occur as was demonstrated in one of the patients in this sample [2]. Packing BIPP impregnated gauze within the cavity and gradually removing it over several weeks has been shown to be effective method in preventing post-operative infections and aiding bone formation at the same time. To our knowledge no other study has reported on this technique in the management of residual cysts.

Primary intraosseous odontogenic carcinoma is described as a squamous cell carcinoma originating within the jaws without primary communication with the oral cavity and arises primarily from rests of odontogenic epithelium [18, 19]. It is a sporadic malignant neoplasm and presents almost entirely in the jaws. The estimated frequency of primary intraosseous odontogenic carcinoma has been reported to be 1–2% of all oral cancer. It has predilection for older males and involves mostly the mandible [18, 19]. Although both cases in this sample occurred in the mandible, one patient was a middle aged female which did not fit the profile of patients affected by this devastating lesion.

Of all odontogenic cysts, radicular/residual cysts have the highest potential for malignant transformation of their epithelium [18]. In a review by Bodner et al., the authors reported that residual⁄radicular cysts were the most frequently (60%) transformed cysts into squamous cell carcinoma, followed by dentigerous cysts (16%) and odontogenic keratocysts (14%). This further highlights the importance of performing a biopsy and managing these inconspicuous appearing lesions [18].

The pathogenesis of malignant transformation of odontogenic cyst epithelium remains unknown. Some authors proposed that the prolonged chronic inflammation may be a precursor factor for malignant change in the cyst epithelium [18]. This finding is supported by the presence of chronic infiltrate of lymphocytes and plasma cells in the cystic lining of malignant cyst epithelium [18].

When primary intraosseous carcinoma is detected in the cyst lining, then the management approach should be selected based on the extent of the lesion [18]. If the carcinoma is within the cyst and has not invaded the adjacent bone, additional surgical management must be avoided and the patient should be monitored regularly. If the margins of the lesion are positive or there is carcinoma invading the surrounding bone as in both our cases in this sample, additional management is indicated. This entails combination of resection, radiation therapy, and chemotherapy with neck dissection if indicated [18].

Due to the retrospective nature of this study, some patients’ records were incomplete while other patients were lost to follow-up and had to be excluded from the study. Another limitation of this study was that only few patients underwent advanced imaging due to the high costs involved and limited access. This did not allow for the study of these lesions using these advanced imaging modalities which can aid in diagnosis and management.

## 5. Conclusion

Residual cysts presented with similar clinico-pathological features as more aggressive cystic lesions in the jaws. They should be highly suspected on the presentation of cystic lesions in edentulous jaws of elderly patients. Biopsy and histopathological examination are the main methods of accurate diagnosis along with radiological correlation. Enucleation is an appropriate surgical method for management of residual cyst with a very low recurrence rate.
